# Identifying functions and prognostic biomarkers of network motifs marked by diverse chromatin states in human cell lines

**DOI:** 10.1038/s41388-019-1005-1

**Published:** 2019-09-19

**Authors:** Li Wang, Hongying Zhao, Jing Li, Yingqi Xu, Yujia Lan, Wenkang Yin, Xiaoqin Liu, Lei Yu, Shihua Lin, Michael Yifei Du, Xia Li, Yun Xiao, Yunpeng Zhang

**Affiliations:** 1 College of Bioinformatics Science and Technology, Harbin Medical University, 150081 Harbin, China; 2 Department of Ultrasonic medicine, The First Affiliated Hospital of Heilongjiang University of Chinese Medicine, 150040 Harbin, China; 3 Weston High School of Massachusetts, 444 Wellesley street, Weston, MA 02493 USA

**Keywords:** Cancer, Biomarkers

## Abstract

Epigenetic modifications play critical roles in modulating gene expression, yet their roles in regulatory networks in human cell lines remain poorly characterized. We integrated multiomics data to construct directed regulatory networks with nodes and edges labeled with chromatin states in human cell lines. We observed extensive association of diverse chromatin states and network motifs. The gene expression analysis showed that diverse chromatin states of coherent type-1 feedforward loop (C1-FFL) and incoherent type-1 feedforward loops (I1-FFL) contributed to the dynamic expression patterns of targets. Notably, diverse chromatin state compositions could help C1- or I1-FFL to control a large number of distinct biological functions in human cell lines, such as four different types of chromatin state compositions cooperating with K562-associated C1-FFLs controlling “regulation of cytokinesis,” “G1/S transition of mitotic cell cycle,” “DNA recombination,” and “telomere maintenance,” respectively. Remarkably, we identified six chromatin state-marked C1-FFL instances (HCFC1-NFYA-ABL1, THAP1-USF1-BRCA2, ZNF263-USF1-UBA52, MYC-ATF1-UBA52, ELK1-EGR1-CCT4, and YY1-EGR1-INO80C) could act as prognostic biomarkers of acute myelogenous leukemia though influencing cancer-related biological functions, such as cell proliferation, telomere maintenance, and DNA recombination. Our results will provide novel insight for better understanding of chromatin state-mediated gene regulation and facilitate the identification of novel diagnostic and therapeutic biomarkers of human cancers.

## Introduction

A variety of posttranslation modifications of histones were reported including histone acetylation and methylation. Aberrant histone modification patterns during tumorigenesis frequently occurred and could trigger pathogenic misregulation of gene expression or genome instability [1]. For example, H3 lysine 9 methyltransferases induces the deposition of H3 lysine 9 trimethylation which is a mark associated with transcriptional repression. H3 lysine 36 dimethylation is associated with increased transcription by counteracting PRC2-dependent histone H3 lysine 27 trimethylation (H3K27me3). Likewise, the acetyltransferase can bind to promoter regions or distal enhancer elements to activate gene expression. Histone demethylase LSD1 can remove enhancer-specific histone H3 mono- and di-methylation on lysine 4 (H3K4me1 and H3K4me2) which in turn decreases enhancer activity. Combinations of histone modifications (acetylation or methylation) could defined open or closed chromatin states which provide information about the transcriptional activity and regulatory element function of the associated DNA across human genome [2, 3]. In addition, emerging evidence implicated that the levels of histone modifications changed dynamically across different human cell types and disease status [1]. The epigenetic regulators such as “writers” and “erasers” of epigenetic marks were highly mutated in human cancer. Thus, aberrant histone modification patterns during tumorigenesis frequently occurred and could trigger pathogenic misregulation of gene expression or genome instability.

Network motifs, as building blocks of complex networks, provide a unifying language to describe regulatory networks [4]. They can perform various computational tasks and biological information processing in biological network [5], and have architecture-dependent responses to internal or external regulation signals, which offer dynamic behaviors underlie a specific cellular state [6]. One of the most well-studied motifs is feedforward loops (FFLs) in which a transcription factor (TF) A regulates another one B, and both jointly regulate a gene C. The coherent type-1 FFL (C1-FFL) and the incoherent type-1 FFL (I1-FFL) frequently occur in the biological networks. In the C1-FFL, a TF A activates another TF B and gene C, and TF B activates gene C. In the I1-FFL, the two arms of the FFL act in opposition: TF A activates gene C, but also represses gene C by activating the repressor TF B.

FFL motif is a best design for signal transduction because it excels in the noise-reduction function [7]. Living organisms could utilize FFLs for better survival in fluctuating environments [8]. C1-FFL is capable of filtering noise asymmetrically to have a precise and robust phenotype of a particular trait (or cellular function) [9]. I1-FFL, as a noise-buffering motif, can facilitate adaptive tuning of gene expression through modulation of TF binding affinities [10]. FFLs are involved in many important biological processes. For example, a positive FFL *IL-6*/*JAK*/*Stat3*, in which *IL-6* activates *JAK* and *STAT3* was involved in tumor proliferation, tumor microenvironment shaping, and metastasis [11]. Disruption of *STAT3* can promote the apoptosis in human cancer cells.

The close cooperation between TF regulations and chromatin modifications gives rise to an interesting question—how regulatory network collaborate with multiple chromatin states for controlling gene expression. To address this question, we collected 269 ChIP-seq data including 140 TFs and genome-wide chromatin states across human cell lines, and then constructed directed regulatory networks with nodes and edges labeled with multiple chromatin states. We characterized the association of diverse chromatin states and network motifs. Diverse chromatin states compositions of targets in C1-FFL or I1-FFL control a large number of distinct biological functions. Furthermore, we identified six chromatin state-marked C1-FFL instances that could act as prognostic biomarkers of acute myelogenous leukemia (LAML). Our results suggest that multiple chromatin states play crucial roles in controlling distinct biological functions of regulatory networks and the modified FFLs could serve as important prognostic biomarkers in human cancers.

## Results

### Integrating directed regulatory networks and chromatin states

**Fig. 1:**
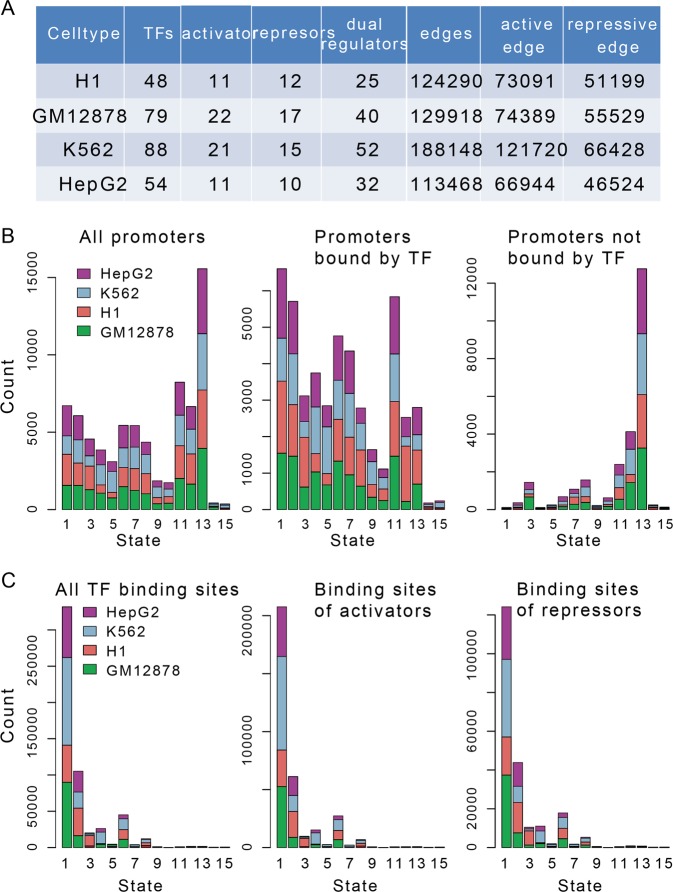
Directed regulatory networks and their chromatin states in four cell lines. **a** The numbers of regulatory interactions and transcription factors from ChIP-seq in four cell types. **b** Fifteen chromatin states used in this study from Ernst et al. The distributions of chromatin states for gene promoters (**c**) and TFBSs (**d**). Colors represent different cell lines

By integrating multiomics data of embryonic stem cells (H1-hESC), lymphoblastoid (GM12878), myelogenous leukemia (K562), and hepatocellular carcinoma (HepG2), we constructed chromatin state-marked transcriptional regulatory network, in which nodes and edges were assigned with specific chromatin states in each of the four cell lines. In the directed transcriptional regulatory network, there are 15 types of chromatin states of genes, which are defined by combinations of histone marks (acetylation or methylation). For example, the active/weak promoter state is characterized by combinations of active epigenetic marks, such as H3K4me2, H3K4me3 and H3K9ac, poised promoter state by both active mark H3K4me3 and repressed mark H3K27me3. The strong/weak enhancer state is characterized by combinations of H3K4me1 and H3K27ac. Different chromatin states provide the central role of chromatin in mediating regulatory signals and controlling DNA access. The number of regulatory interactions ranged from 113,468 to 188,148 in the four cell lines (Fig. 1a).

We found that regulators and targets within regulatory networks showed diverse chromatin states, reflecting their active or repressed states. Apart from the active promoters, a number of repressed states of genes present in regulatory networks were also observed (Fig. 1b). One possible explanation is that TF binding is required for heterochromatin formation [12]. However, the promoters of genes not connected to regulatory networks are highly enriched for silent chromatin states (e.g., heterochromatin state). Only a few chromatin states were represented on local TF binding sites (TFBSs), primarily focusing on active and weak promoter states as well as a minority of enhancer states (Fig. 1c).

### Characterizing diverse chromatin states of network motifs

**Fig. 2:**
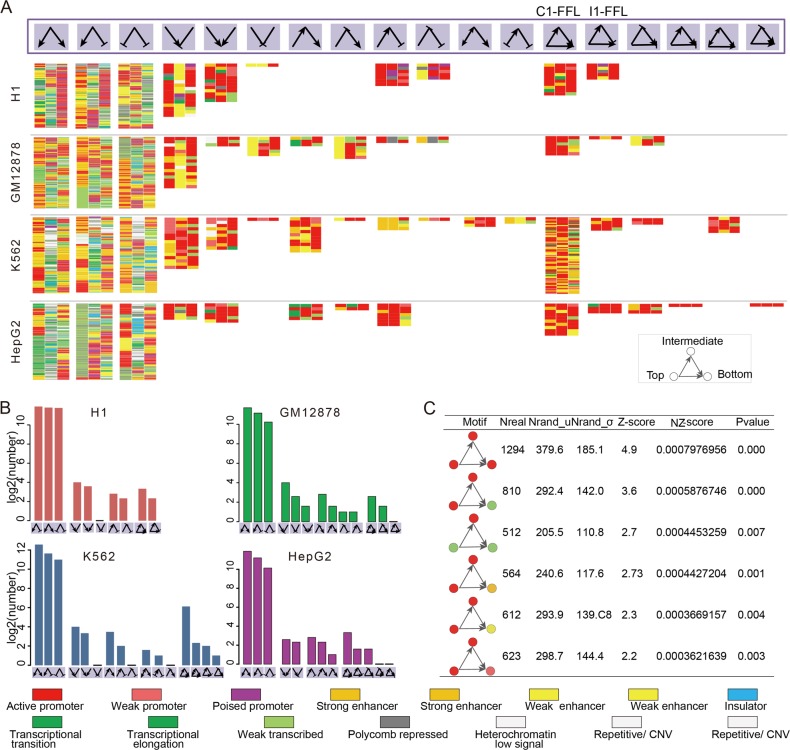
Characterizing diverse chromatin states of network motifs. **a** Significant association between chromatin state compositions and total eighteen types of motifs in four cell lines. Different colors indicate fifteen chromatin states used in this study from Ernst et al. **b** The numbers of chromatin state compositions associated with a particular motif. **c** Six kinds of enriched chromatin state-modified C1-FFL motifs in GM12878. The colors of nodes indicate the chromatin states of corresponding genes

To gain insights into dynamic association between chromatin states and regulatory networks, we sought to systematically search for three-node motifs by taking into account both the network topological structures and chromatin states of nodes and edges. Comparing with random networks, we determined the over-represented association between three-node motifs and gene chromatin states, which were defined as chromatin state-marked network motifs. We found a large number of network motifs marked by diverse chromatin state compositions in the four cell types, including 10,974 in H1, 6,918 in GM12878, 11,340 in K562, and 7349 in HepG2, referring to a total of eighteen types of motif structures (Fig. 2a). We observed that the single-input module circuits were connected with a large cluster of chromatin state compositions (Fig. 2b). FFLs, one of the most important network motifs, also marked by diverse chromatin states and were consistently present in all of these cell lines, especially the C1-FFL and I1-FFL. For instance, in GM12878, C1-FFLs cooperated with six kinds of chromatin state compositions were significantly enriched (Fig. 2c). For example, an active promoter state-marked C1-FFL (Fig. 2c) represents that each of three nodes is both marked by active promoter state which defined by combinations of high level of H3K4me2, H3K4me3, and H3K9ac. Active promoter chromatin state represents open chromatin conformation and high DNA accessibility, which in turn facilitate TF binding and activates gene expression [13]. Thus, chromatin state of genes could help FFL motif to precise control gene expression, signal transduction and biological information-processing functions. Our results underline the extensive association between chromatin states and regulatory networks, suggesting that only focusing on network structures are not enough to uncover complex regulation principles underlying expression dynamics.

### Diverse chromatin states influencing target expression in FFLs

**Fig. 3:**
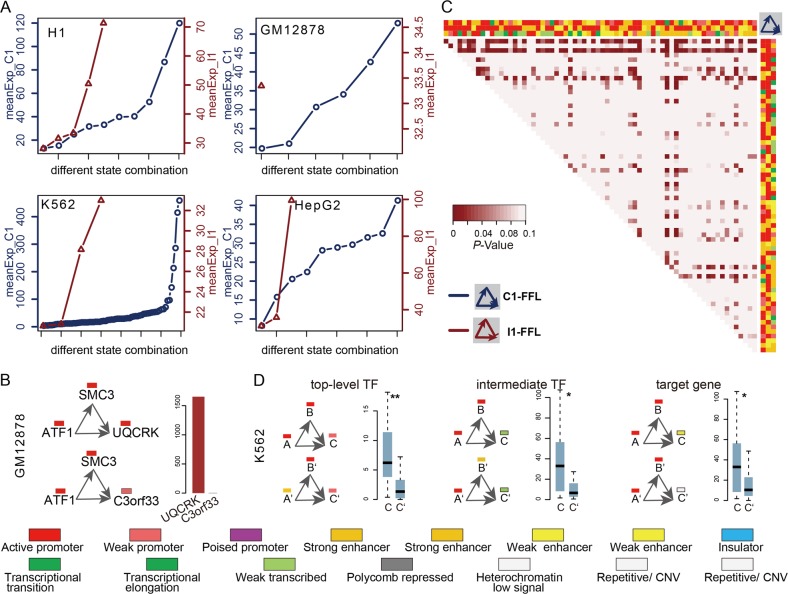
Diverse chromatin states influencing target expression in FFLs. **a** The mean expression levels of targets in C1-FFL (blue) or I1-FFL (red) marked by different chromatin states in four cell lines. **b** Examples of different chromatin states at bottom position of C1-FFL can lead to expression differences of target genes. **c** Significant expression differences of targets between different chromatin state compositions associated with C1-FFL in K562 using Wilcoxon’s rank sum test. **d** Examples of different chromatin states at different positions of C1-FFL can lead to expression differences of target genes

**Fig. 4:**
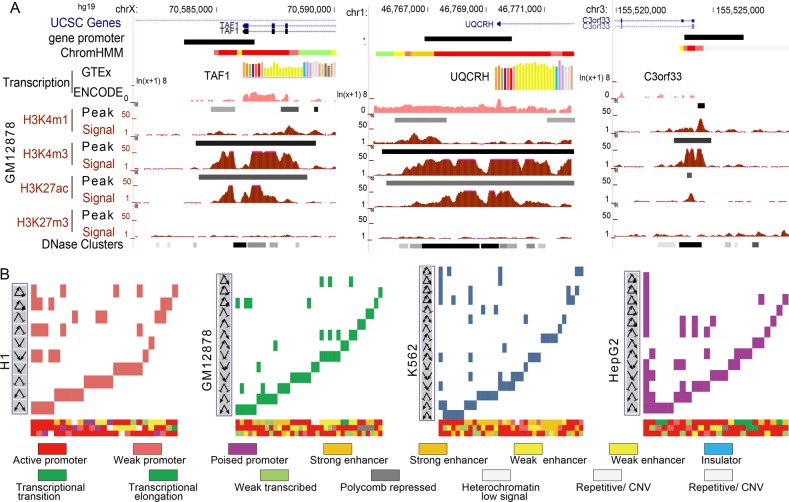
Diverse chromatin states influencing target expression and functions of FFLs. **a** The distribution of gene expression, chromatin state, and histone modifications of gene promoters in GM12878. **b** The distribution of the top five chromatin state compositions with the highest frequency across different types of motifs in each cell line

We analyzed expression levels of target genes in FFL instances labeled with diverse chromatin states. Interestingly, a dynamic expression change of target genes modified by diverse chromatin states is observed in most cell lines, even those sharing the same motif structure (Fig. 3a). For example, the promoters of *TAF1* and *SMC3* (active state) are marked by active epigenetic marks H3K4me3 and H3K27ac and showed high-level expression of *TAF1* and *SMC3*. *TAF1* and *SMC3* together regulate two targets *UQCRH* (active state) and *C3orf33* (weak active state) forming two C1-FFL (Fig. 3b). Especially, a range from 2.9 to 458.8 FPKM were observed in 69 types of chromatin state compositions which significantly modified the C1-FFL in K562. We found significant expression differences between diverse chromatin state compositions associated with C1-FFL in K562 (Fig. 3c). Furthermore, chromatin state alterations at different positions of FFLs can lead to expression differences rather than just epigenetic states of target genes (Fig. 3d). The *UQCRH* showed enrichment of H3K27ac and H3K4me3 marks and high-level expression. The decreased active epigenetic marks of *C3orf33* significantly decreased its expression (Fig. 4a). In K562, “active promoter” and “strong enhancer” states occurred at top and intermediate positions of C1-FLL had significantly different expression of target genes. These observations highlight the importance of diverse chromatin states in FFLs to finely regulate expression of target genes.

### Functions and prognostic utility of FFLs marked by diverse chromatin states

We were interested in determining whether diverse chromatin states were frequently used by different motifs in a given cell line. For each over-represented motif structure, the top five state compositions were used to display the usage of chromatin states across different motif structures. We found that different types of motifs were associated with diverse chromatin states and a few state compositions shared by multiple motifs in each cell line (Fig. 4b).

**Fig. 5:**
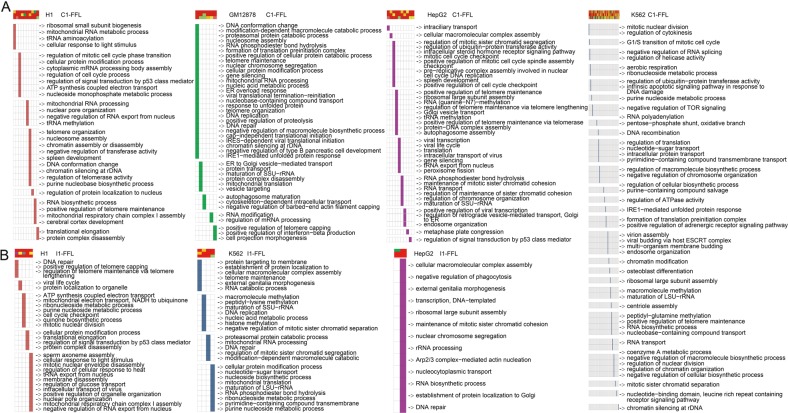
Revealing functions of FFLs marked by diverse chromatin states. The significantly enriched biological processes using target genes of C1-FFL (**a**) and I1-FFLs (**b**) marked by diverse chromatin states in four cell types

The diverse chromatin states gives rise to an important question, that is, why different chromatin states are required for a given motif in human cell lines. To solve this problem, we performed functional enrichment analysis using targets of FFL with a specific chromatin state composition in the four cell types. We found that these targets were significantly enriched in many important biological functions, such as cell cycle checkpoint, DNA repair and regulation of telomere maintenance (Fig. 5a for C1-FFL; Fig. 5b for I1-FFL). Notably, diverse chromatin state compositions of targets in C1-FFL or I1-FFL contributed to a large number of distinct biological functions in human cell lines. For instance, in GM12878, “active promoter” states linking with C1-FFL control biological functions associated with DNA replication and DNA repair, however, another chromatin state composition (top position with “active promoter” state, intermediate- and bottom positions with “transcription elongation” states) was related to autophagosome maturation (Fig. 5a). Five types of chromatin state compositions modifying H1-associated I1-FFL were related to different biological functions, such as DNA repair, cycle checkpoint, protein location to organelle, regulation of signal transduction by *p53* class mediator and regulation of RNA export from nucleus, respectively (Fig. 5b).

**Fig. 6:**
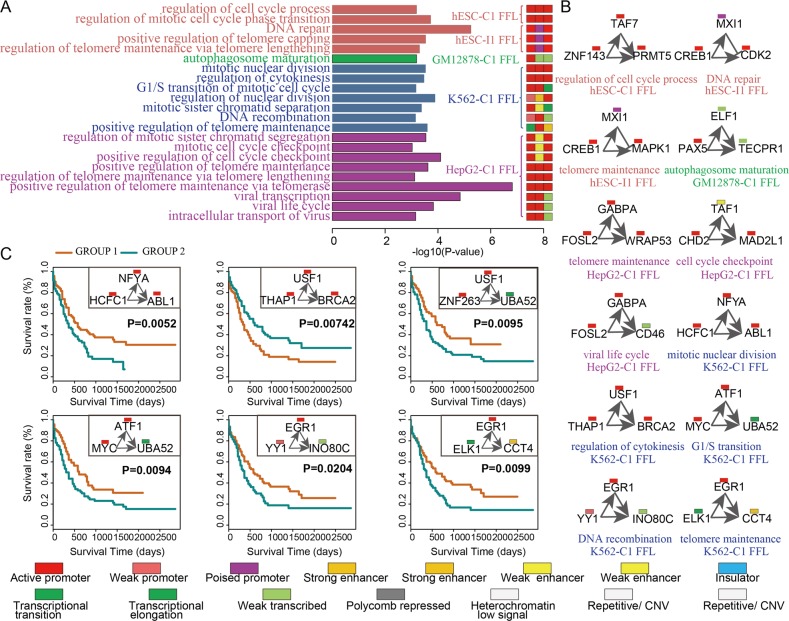
Diverse chromatin states contributing to distinct functions and prognosis. **a** The examples of GO biological functions (left) significantly enriched by FFL targets, which are marked by diverse chromatin states (right). **b** Examples of FFL instances marked by a specific chromatin state composition and their associated biological functions. **c** Kaplan–Meier survival plots of overall survival using K562-associated C1-FFL instances. AML patients were divided into two groups according to the median of a linear combination of expression values of three node in FFLs. Group 1 (yellow curve) and group 2 (blue curve) indicate the high and low expression of an FFL, respectively

Notably, diverse chromatin states seem to be associated with distinct biological functions. Importantly, these functions are related to specific cellular context (Fig. 6a). As an example, H1-associated C1-FFL (*TAF7*-*ZNF143*-*PRMT5*) marked by “active promoter” states in which *TAF7* together with *ZNF143* regulate protein arginine *PRMT5* contributing to functions associated with the self-renew of stem cell, such as regulation of cell cycle process (Fig. 6a, b). *PRMT5* acting as a epigenetic regulator is required for human embryonic stem cell proliferation [14]. Inhibition of methyltransferase *PRMT5* suppresses self-renewal of human leukemia stem cells [15]. Two H1-associated I1-FFLs (*CREB1*-*MXI1*-*CDK2* and *CREB1*-*MXI1*-*MAPK1*) marked by “active promoter” (top), “poised promoter,” (intermediate) and “active promoter” (bottom) which, captured functions associated with DNA repair and telomere maintenance (Fig. 5a, b). The *CDK2* plays an important role in DNA damage response in human embryonic stem cells [16]. The “poised promoter” of *MXI1* which is a negative regulator of cell cycle leads to its reduced expression level and in turn releases *MXI1*-mediated inhibition of *CDK2* and mitogen-activated protein kinase 1 (*MAPK1*). Telomere maintenance is associated with stem cell renewal. In particular, it is consistent with essential roles of the “poised promoter” state in stem cell maintenance and subsequent differentiation [17]. In GM12878, *PAX5* (“active promoter”) and *ELF1* (“weak transcribed”) together regulated downstream target *TECPR1* (“weak transcribed”) forming a C1-FFL (*PAX5*-*ELF1*-*TECPR1*) which mediated autophagosome maturation (Fig. 6a, b). Two HepG2-associated C1-FFLs (*FOSL2*-*GABPA*-*WRAP53* and *FOSL2*-*GABPA*-*CD46*) marked by “active promoter” states of *FOSL2*, *GABPA* and *WRAP53*, a weak transcribed state of *CD46* controlling telomere maintenance and viral life cycle, respectively. *WRAP53*, a novel regulator of *p53*, promotes cancer cell survival and is a potential target for cancer therapy [18]. Hepatitis B virus infection is one of major viral risk factors for hepatocellular carcinoma [19]. *CD46* acting as a complement regulatory protein contribute to escape of hepatoma cells from complement-dependent cytotoxicity [20]. Another example C1-FFL (*CHD2*-*TAF1*-*MAD2L1*) marked by an “active promoter” state of *CHD2*, a “weak/poised enhancer” state of *TAF1* and an “active promoter” of mitotic arrest deficient 2 (*MAD2L1*) controlling cycle cell checkpoint [21].

More importantly, we try to identify prognostic biomarkers in terms of chromatin state-marked network motifs in human cancers. K562-associated FFLs marked by diverse chromatin states were used to divide patients with LAML into high-risk and low-risk groups. We found that six K562-associated FFLs marked by diverse chromatin states could distinguish the LAML patients with different survival times (Fig. 6c and Table 1). For example, two K562-associated C1-FFLs (*HCFC1*-*NFYA*-*ABL1* and *THAP1*-*USF1*-*BRCA2*) marked by “active promoter” states captured functions associated with nuclear division and regulation of cytokinesis, respectively (Fig. 5a, b). Mutations of HCFC1 gene are frequently observed in individuals with LAML [22]. NFYA could promote self-renewal of hematopoietic stem cell and inhibition of NFYA expression could hinder the progression of endometrial cancer [23]. Fusion of ABL1 to BCR/TEL/NUP214 is observed in a large number of leukemia patients and allosteric stimulation of the normal ABL1 kinase activity enhanced the antileukemia effect of ABL1 tyrosine kinase inhibitors [24]. Low expression of *HCFC1*-*NFYA*-*ABL1* correlated with patient’s poor prognosis (*P*-value = 5.2e−3, log-rank test, Fig. 6c). THAP1, a gene encoding a nuclear proapoptotic protein, could induce cellular apoptosis in acute lymphoblastic leukemia cells [25]. Tumor suppressor protein BRCA2 paly important role in DNA double-strand break repair. BRCA2 deficiency may predispose leukemia cells to synthetic lethality triggered by PARP1 inhibitors [26]. High expression of *THAP1*-*USF1*-*BRCA2* correlated with patient’s poor prognosis (*P*-value = 7.4e−3, Fig. 6c). Likewise, the expression of two K562-associated C1-FFLs (*ZNF263*-*USF1*-*UBA52* and *MYC*-*ATF1*-*UBA52*) was positively associated with the longer overall survival of AML patients (*P*-value = 9.5e−3 and 9.4e−3, respectively, Fig. 6c). Both of these FFLs were associated with G1/S transition of mitotic cell cycle in which top and intermediate TFs were associated with “active promoter” states and bottom target with a “transcriptional elongation” state. Oncoprotein MYC is required for chronic myelogenous leukemia progression [27]. Another example was DNA recombination-associated CI-FFL *YY1-EGR1*-*INO80C* in which *YY1* with a “weak promoter” state directly binds to the *EGR1* promoter with an “active promoter” state for its transactivation. Both of them regulate INO80C, a subunit of chromatin remodeling complex (INO80 complex) showing a “weak transcribed” state, forming a C1-FFL controlling DNA recombination in K562. Low expression of *YY1-EGR1*-*INO80C* was significantly correlated with AML patient’s poor prognosis (*P*-value = 0.02, Fig. 5c). *ELK1* (Transcriptional transition) induced and rapidly regulate *EGR1* transcription (active promoter) [28], both together induced downstream target *CCT4* (strong enhancer) forming a C1-FFL in K562 (*ELK1*-*EGR1*-*CCT4*), which mediated positive regulation of telomere maintenance. ELK1 could promote osteosarcoma progression by the inactivating Hippo pathway [29]. Loss of EGR1 in cooperation with TP53 and APC loss could result in myeloid neoplasms [30]. Amplification of CCT4 gene were detected in clinical lung cancer cases and associated with decreased survival [31]. The low expression of a C1-FFL (*ELK1-EGR1*-*CCT4*) correlated with patient’s poor prognosis (*P*-value = 9.9e−3, log-rank test, Fig. 6c). The chromatin state-marked FFL is involved in a mechanism that maintains the length and integrity of telomeres which as an independent prognostic factor in chronic lymphocytic leukemia [32]. It is consistent with previous results that telomere maintenance can act as a target for anticancer drug discovery and its disruption triggers cell death [33]. These observations suggest different chromatin states could modulate TF binding and help FFL motif to precise control gene expression and distinct functions. We identified six chromatin state-marked FFLs which could act as independent prognostic factors of leukemia. These chromatin state-marked FFL circuit play key roles in identifying prognostic biomarkers and understanding underlying mechanism for the pathogenesis of human cancers.

**Table 1:** The chromatin states and functions of FFL signatures

C1-FFLs in K562			Chromatin state of genes			Functions
Top TF	Inter-TF	Target	Top TF	Inter-TF	Target	
HCFC1	NFYA	ABL1	Active promoter	Active promoter	Active promoter	Mitotic nuclear division
THAP1	USF1	BRCA2	Active promoter	Active promoter	Active promoter	Regulation of cytokinesis
ZNF263	USF1	UBA52	Active promoter	Active promoter	Transcriptional elongation	G1/S transition of mitotic cell cycle
MYC	ATF1	UBA52	Active promoter	Active promoter	Transcriptional elongation	G1/S transition of mitotic cell cycle
YY1	EGR1	INO80C	Weak promoter	Active promoter	Weak transcribed	DNA recombination
ELK1	EGR1	CCT4	Transcriptional transition	Active promoter	Strong enhancer	Positive regulation of telomere maintenance

## Discussion

We constructed regulatory networks labeled with chromatin states in human cell lines and performed a systemic analysis of network motifs marked by chromatin states. We found that dynamic association between diverse chromatin states and network motifs. Diverse chromatin states could help regulatory network to control distinct biological functions that are essential for cell identity in human cell lines. Notably, we identified six chromatin state-marked FFL signatures as network-based prognostic biomarkers in LAML. The detection of FFL motifs will help understanding the mechanism of transcriptional regulation and network evolution [34]. Previous Studies showed that FFL motif may represent evolutionary conserved topological units of cellular network and exhibited high frequencies and conserved across mouse and human cell/tissue type regulatory networks as well as dominance networks from data published over the past 80 years [35–38]. Recently, ConsHMM, an extesion of ChromHMM, is presented to define de novo “conservation states” based on the combinatorial and spatial patterns of a multiple species DNA sequence alignment [39]. Analysis of relationship of conservation states to chromatin states showed that almost all of chromatin states were enriched for at least one of conservation states [39], suggesting a high evolutionary conservation of chromatin states. Therefore, it is important to analyze the evolutionary conservation of chromatin state-marked FFL motifs in human diseases. Our approach depends on multi-dimensional data availability including gene expression, chromatin states, TFs Chip-seq and clinical data to identify prognostic chromatin state-marked FFL biomarkers. However, the lack of large-scale multidimensional omics and clinical data of human cancers limits the analysis of evolutionary conservation of chromatin state-marked FFL motifs. As more large-scale multidimensional omics and clinical data of human cancers become available, it could further improve robustness and predictive capacities and extend the application of our approach.

In addition, in order to validate the stability of positive or negative relationships between TFs and their targets, Spearman’s rank correlation coefficient is calculated between a TF and a target using Human Body Map 2.0 Project RNA-seq data for 16 different human tissues. As a comparison, we found a high consistency between the results from the Spearman’s rank correlation coefficient and those from Pearson correlation coefficient (PCC), with an average of 82.6% (82.2% for H1, 82.8% for GM12878 81.3% for HepG2, and 84.0% for K562). Furthermore, in order to validate the effectiveness of the method, we used the FANMOD approach [40], a tool for colored motif detection in colored networks, to re-search the association between network motifs and chromatin states. In order to compare with the FANMOD approach, we removed chromatin states of edges from the directed regulatory networks and then used FANMOD to re-search the association between network motifs and chromatin states using the same thresholds. As a comparison, we found a high consistency between the results from the FANMOD approach and those from our method, with an average of 84.64% (100% for H1, 83.33% for GM12878, 70.59% for K562).

We systematically examined the association between epigenetic regulation and transcriptional regulation in human cell lines. Our results underline important roles of diverse chromatin states in fine regulation of gene expression and distinct biological functions. Remarkably, we identified six chromatin state-marked FFLs acting as important prognostic biomarkers of LAML. Chromatin state-mediated FFLs will provide novel insight for the identification of novel diagnostic and therapeutic biomarkers of human cancers.

## Materials and methods

### Data sets

ChIP-seq dataset: we obtained 269 ChIP-seq data sets referring to 140 TFs in H1, GM12878, K562, and HepG2 from ENCODE (GSE32465 and GSE31477) [41]. Reads from TF ChIP-seq data were aligned to hg19/GRCh37 assembly of the human genome using Bowtie2 (version 2.2.3) [42] allowing up to two mismatches. Only uniquely mapping reads were retained and multiply mapping reads were discarded. The MACS2 [43] peak caller was used to compare ChIP-seq signal with a corresponding whole cell extract sequenced control to identify regions of ChIP-seq enrichment (peaks) at the threshold of *P* < 10^−5^ [44]. MACS2 can identify peaks from ChIP-seq data and reports the summits of peaks (±20 bp) as TFBSs [45].

DNase-seq dataset: for each of these cell lines, sequence reads of DNaseI-seq experiments from ENCODE Project Consortium [41] were mapped to human reference genome versions of hg19/GRCh37 using the Bowtie aligner, allowing a maximum of two mismatches. Only reads mapping uniquely to the genome were used in the analyses. DNaseI hypersensitive sites (DHSs), which provide information about which regions of a promoter have open chromatin [46], were identified using the Hotspot algorithm at an false positive rate (FDR) of 1% [47], and were downloaded from the UCSC genome browser.

RNA-seq dataset: we extracted whole cell long polyA-selected RNA-seq data sets from ENCODE (GSE26284) [41]. Moreover, RNA-seq data sets of 16 human tissues (adipose, adrenal, brain, breast, colon, heart, kidney, liver, lung, lymph node, ovary, prostate, skeletal muscle, testes, thyroid, and white blood cells) were extracted from Human Body Map 2.0 Project (GSE30611; 50-nt paired end reads, 2018) [48]. In RNA-seq analyses, the raw reads were aligned using the TopHat (version 2.0.11) to the human reference genome (hg19/GRCh37), allowing a total of two mismatches [49]. Fragments per kilobase of exon per million mapped reads (FPKM) of gene-level expression of UCSC known genes were calculated using Cufflinks (version 2.2.1) [48]. FPKM method can provide a length and depth normalization to permit both within-sample and cross-sample comparisons [50].

### Constructing directed regulatory networks in human cell lines

#### Identifying TF-gene regulatory interactions

UCSC hg19 Known Gene annotation and 3 kb promoters (2.5-kb upstream to 0.5-kb downstream) were used [51]. For each cell type, a gene was considered a target of a TF if it had at least one TFBS in its promoter region that overlapped with a DHS by at least one base pair [52]. By combining regulatory interaction between TFs and targets, a TF-gene regulatory network in a particular cell line was established.

#### Determining positive or negative relationships between TFs and their targets

PCC was calculated between a TF and a target using Human Body Map 2.0 Project RNA-seq data for 16 different human tissues. Positive relationships between a TF and their targets were determined by positive PCCs, and vice versa [53].

#### Identifying chromatin states of gene promoters and TFBS

We obtained genome-wide 15 types of chromatin states based on recurrent combinations of histone marks using a multivariate Hidden Markov Model (HMM) for these four cell lines from UCSC genome browser [3]. Firstly, chromatin states associated with a specific gene promoter are obtained using genome-scan method according to the chromosome and the position on the chromosome. Secondly, in order to determine which chromatin state is the most associated to the specific gene, the fold enrichment method was used [54]. In detail, the enrichment score *S* of a type of chromatin state *s* on a given region (such as a promoter or a TFBS) was calculated by: $S = \frac{{r_s}}{n}/\frac{{c_s}}{t}$. Where *r*_*s*_ represents the number of bases in a given region overlapping with a specific chromatin state *s* (*s* in 1:15), *n* represents the number of bases in the region, *c*_*s*_ represents the total number of bases of the chromatin state *s* and *t* represents the total number of bases of all chromatin states. A chromatin state with the highest enrichment score was selected as the chromatin state of a promoter or a TFBS [54].

### Identifying significant association between chromatin state and network motifs

We mapped the chromatin states of gene promoters and TFBS to the nodes (i.e., TFs and genes) and edges (i.e., TF-gene regulatory interactions), respectively, for forming the directed regulatory network labeled with chromatin states. In order to identify over-represented chromatin state-marked network motifs, frequency of occurrence of a specific three-node network subgraph in which nodes marked by specific chromatin states was estimated in the real network. Next, the randomized networks were used to calculate the significance level of the specific chromatin state-marked network subgraph. A degree-preserving randomized network was obtained by swapping edges between random pairs of nodes 10^6^ times, keeping the same number of appearances of all two-node subgraphs as in the real network. It can avoid assigning a high significance to a network pattern only because it contains a highly significant subpattern. We repeated this procedure 1000 times to generate 1000 randomized networks, and assigned a P-value to the chromatin state-marked network subgraph as the fraction of randomized networks that lead to a greater or equal number of frequency than those observed in the real network. Besides, *z*-score was calculated by observed frequency of the chromatin state-marked subgraph appears in the network subtracting the mean of its appearances in the randomized network and dividing by the standard deviation of its appearances in the randomized network. A normalized *z*-score by *z*-scores normalized to length 1 was also used for evaluating the significance as previously proposed [55]. We identified significant association between chromatin states and network motifs according to the following criteria: (i) *P* < 0.01 (ii) normalized *z*-score > 0 [56]. (iii) The number of the motif appeared in the real network should not be <500.

### Prognosis analysis using FFLs marked by diverse chromatin states

Gene expression and survival data of 197 patients with LAML were downloaded from TCGA Data Portal (https://portal.gdc.cancer.gov/) on January 2015. We assigned each patient a risk score according to a linear combination of the expression level of nodes in the FFL instances weighted equally. Patients with higher risk scores are expected to have poor survival outcomes. We next divided the LAML patients into high-risk and low-risk groups using the median risk scores. The Kaplan–Meier method and log-rank test were used to evaluate the effect of FFLs on overall survival.

### Functional analysis

We performed functional enrichment analysis based on Gene Ontology (GO) annotation terms using R package clusterProfile (version: 2.0.0) [57]. We used enrich GO function of clusterProfiler to calculate enrichment test for GO terms based on hypergeometric distribution. The clusterProfiler adjust the estimated significance level using Benjamini–Hochberg method to control the FDR in multiple testing. The adjusted *P*-value < 0.05 were regarded as the cutoff criterion for GO enrichment analysis.
